# 

**Published:** 1894-12

**Authors:** 


### CONTENTS:

ORIGINAL ARTICLES: PAGE

The Effect of Age on the Organs of the Horse. By Prof. J. T. Duncan .... 437
The Value of Prophylactic Treatment in Anthrax. By John Faust, V. S. .... 440
Comparative Pathology. By J. Adami, M.A., M.D. .... 444
A Large Abdominal Hernia Successfully Treated. By E. E. Bittles, V.S. .... 447
A Few Abnormal Results following Dehorning Cattle. By Dr. J. D. Nichbert .... 449
Treatment of Ossific Diseases of the Joints by Hypodermic Injections. By J. H. Wattles, D.V.S. .... 450
The Diagnosis of Tuberculosis, Dealing Especially with the Dose of Tuberculin to be Used in Testing for Tuberculosis. By Prof. F. L. Russell, V.S. .... 453
Dislocation Between the Second and Third Cervical Vertebræ in a Horse, without any Paralysis. By A. W. Clement, V.S. .... 455
The Anatomy of the Large American Fluke (*Fasciola Magna*). By Charles Wardell Stiles, Ph.D. .... 457
Pennslyvania—Proposed Act to Establish "The State Live Stock Sanitary Commission of Pennslyvania" .... 463
Massachusetts—Prevention and Eradication of Bovine Tuberculosis .... 466
Massachusetts Regulations .... 469
The Veterinary Association of Manitoba .... 471

EDITORIAL:

JOURNAL OF COMPARATIVE MEDICINE AND VETERINARY ARCHIVES FOR 1895 .... 473

Roads to Veterinary Education .... 472

Manitoba the Best .... 474

VETERINARY NEWS .... 475

COLLEGE NOTES .... 479

Ontario Veterinary College, Toronto, Canada .... 483
Obituary Notice. Prof. Thomas Walley .... 484

SOCIETY PROCEEDINGS:

Iowa State Veterinary Medical Association .... 485
Keystone Veterinary Medical Association .... 488
Illinois State Veterinary Medical Association .... 489
Proceedings of the Society for the Study of Comparative Psychology in Connection with the Faculty of Comparative Medicine and Veterinary Science of McGill University, Montreal, Canada .... 490

Index .... 503

